# Bridging the Gap in Clubfoot Care: A Framework for Sustainable Rehabilitation in Low-Resource Settings

**DOI:** 10.7759/cureus.107279

**Published:** 2026-04-18

**Authors:** Ayisha Ayub, Areej Fatima

**Affiliations:** 1 Research and Development, Ayesha Bashir Hospital, Gujrat, PAK

**Keywords:** clubfoot, clubfoot model, congenital talipes equinovarus, disability reduction, low- and middle-income countries, rehabilitation, replicable model

## Abstract

Clubfoot is one of the most common congenital musculoskeletal deformities and remains a major cause of preventable childhood disability in low- and middle-income countries. In Pakistan, access to timely, standardized clubfoot care is limited, particularly outside major urban centers, resulting in delayed treatment and preventable lifelong disability. This technical report describes the development, evolution, and operational components of a sustainable clubfoot care and rehabilitation model established in Gujrat, Pakistan, beginning in 2010. The program transitioned from a single-room outpatient clinic into a comprehensive rehabilitation hub delivering Ponseti-based correction, prosthetic and orthotic services, physiotherapy, and structured long-term follow-up. Over 2,000 patients were enrolled, generating approximately 20,000 cumulative patient encounters. The model emphasizes phased infrastructure development, locally trained multidisciplinary workforce capacity, longitudinal service delivery, and financial sustainability through hybrid social enterprise mechanisms. Functional outcomes included independent ambulation, school participation, pain-free mobility, and workforce reintegration. This framework demonstrates that sustainable disability reduction in resource-constrained settings can be achieved through locally led, capacity-aligned system development and may serve as a replicable model for similar contexts.

## Introduction

Congenital talipes equinovarus, commonly referred to as clubfoot, affects approximately 1.24 per 1,000 live births worldwide and represents one of the leading causes of preventable physical disability in children [1]. The burden is disproportionately concentrated in low- and middle-income countries (LMICs), where nearly 80% of affected children do not receive timely treatment due to limited awareness, delayed diagnosis, and restricted access to standardized care systems [2]. In Pakistan alone, an estimated 7,500 children are born annually with clubfoot, constituting a substantial yet under-addressed public health challenge [1].

Untreated or inadequately treated clubfoot frequently progresses to neglected deformity, characterized by rigid equinovarus positioning, pain, abnormal gait mechanics, and eventual social and economic marginalization. The Ponseti method has been widely established as the gold-standard treatment for idiopathic clubfoot due to its high success rates, low cost, and suitability for low-resource environments [3]. Despite this, service provision in many regions remains fragmented and urban-centered, limiting equitable access for peri-urban and rural populations.

In 2010, a structured clubfoot care program was established in Gujrat, Pakistan, evolving into a comprehensive rehabilitation model integrating clinical care, orthotic services, physiotherapy, and long-term follow-up. This descriptive programmatic observational report includes over 2,000 patients and approximately 20,000 encounters. Approximately 90% achieved plantigrade correction, with a relapse rate of ~40% and tenotomy required in most cases. Outcomes were assessed through clinical follow-up, supported by Pirani scoring where available. While standardized outcome measurement was not uniformly available, the model demonstrates a feasible and potentially scalable approach to clubfoot rehabilitation in resource-limited settings.

## Technical report

Methods

This study is a descriptive programmatic observational report based on longitudinal service delivery data from a clubfoot rehabilitation program established in Gujrat, Pakistan, since 2010. Regular clinical services were used to enroll patients without randomization or predetermined sampling. Both idiopathic and neglected clubfoot cases from both adult and pediatric age groups were covered in the program. Clinical assessment was routinely performed using the Pirani scoring system during treatment; however, complete longitudinal datasets were not consistently available for all patients. Data were collected retrospectively from program records, including patient volume, treatment interventions (e.g., casting, tenotomy), number of visits, and service utilization patterns. Outcomes were evaluated via routine clinical follow-up and programmatic observation, including correction status, ambulation ability, brace compliance, relapse occurrence, and functional reintegration. Due to variability in data completeness over time, outcomes are presented using a combination of descriptive and available quantitative indicators. Further details about the center and its services can be accessed at: https://www.facebook.com/p/Club-Foot-Center-Gujrat-Pakistan-100083358638086/

Program design

The establishment of the clubfoot care center in Gujrat began with the recognition of a clear service vacuum in the region. At inception, the program operated within the existing infrastructure of a local welfare organization, utilizing a single outpatient room to initiate Ponseti-based non-surgical treatment. This “minimum viable facility” approach minimized initial capital expenditure while allowing immediate service delivery to an underserved population. From the outset, the model was intentionally designed to move beyond short-term, charity-based care toward structured, long-term system development. Public health awareness campaigns were introduced to facilitate early identification and referral.

Service delivery model

Standardized clinical care was implemented through consistent adherence to Ponseti protocol principles, including serial casting, percutaneous tenotomy when indicated, and brace compliance monitoring. Holistic rehabilitation was incorporated through orthotic support, physiotherapy, and structured follow-up mechanisms to ensure sustained correction and functional mobility. The patient population included infants, children, and a smaller proportion of older individuals presenting with neglected deformities. Both complicated and idiopathic situations were handled.

Infrastructure development and expansion

**Figure 1:**
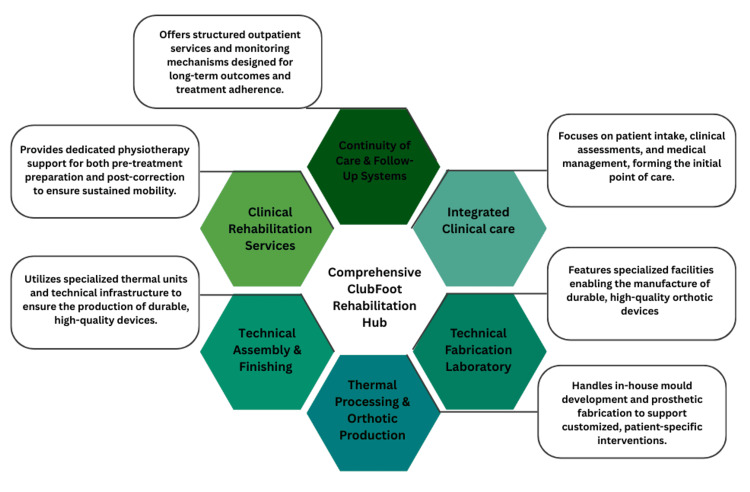
Integrated functional components of a comprehensive clubfoot rehabilitation hub. The model demonstrates how coordinated clinical care, in-house technical fabrication, rehabilitation services, and longitudinal follow-up are delivered within a single integrated system. The framework is informed by programmatic experience and supported by service delivery data. Image credit: Image created by the authors using Canva (Canva Pty Ltd., Sydney, Australia).

Infrastructure expansion occurred incrementally and was directly linked to service demand and clinical stability rather than predetermined construction targets. Surgical support was accessed through partnerships with local hospitals when necessary. As patient volumes increased, the service transitioned into a dedicated facility comprising integrated clinical, technical, and rehabilitative units (Figure 1).

Workforce development

A central pillar of the program’s sustainability was the development of a locally trained multidisciplinary workforce. Clinical leadership oversaw Ponseti management and surgical referrals, technical specialists managed in-house prosthetic and orthotic fabrication, rehabilitation professionals supported functional recovery and social reintegration, and operations teams ensured continuity of care and follow-up adherence. Institutional partnerships with national and international organizations provided structured training and quality oversight while preserving local governance.

Service expansion pathway

**Figure 2:**
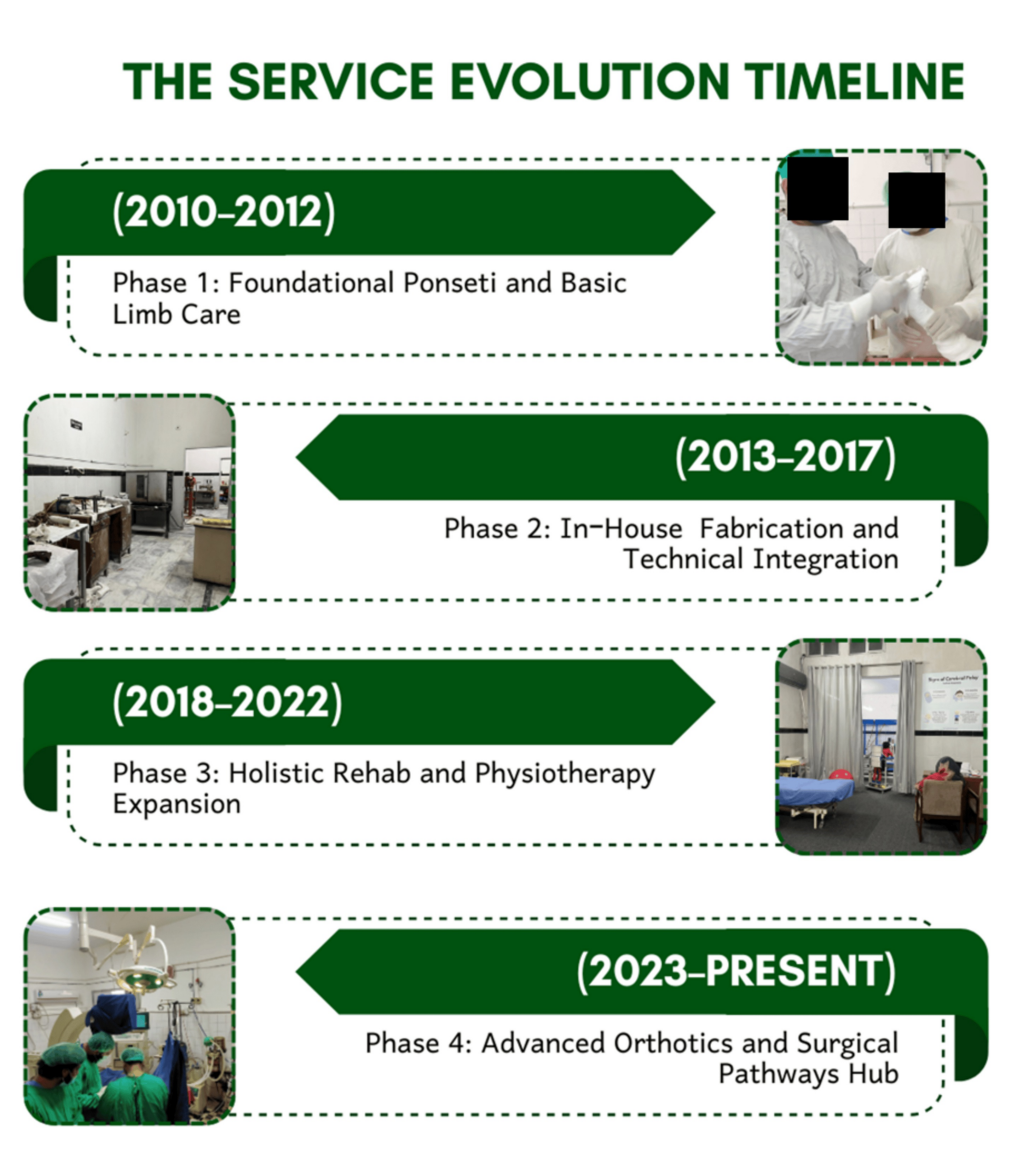
Phased evolution of the clubfoot care program. This figure illustrates the progression from a basic Ponseti-based clinic to a comprehensive rehabilitation model over time. Representative images corresponding to each phase are included to provide real-world context and demonstrate infrastructure and service development across the program lifecycle. Image credit: Image created by the authors using Canva (Canva Pty Ltd., Sydney, Australia).

Service expansion followed a phased evolution model in which casting services were introduced first, followed by bracing, physiotherapy integration, neglected clubfoot management, and adult deformity rehabilitation. This progressive expansion occurred over more than a decade (Figure 2).

**Figure 3:**
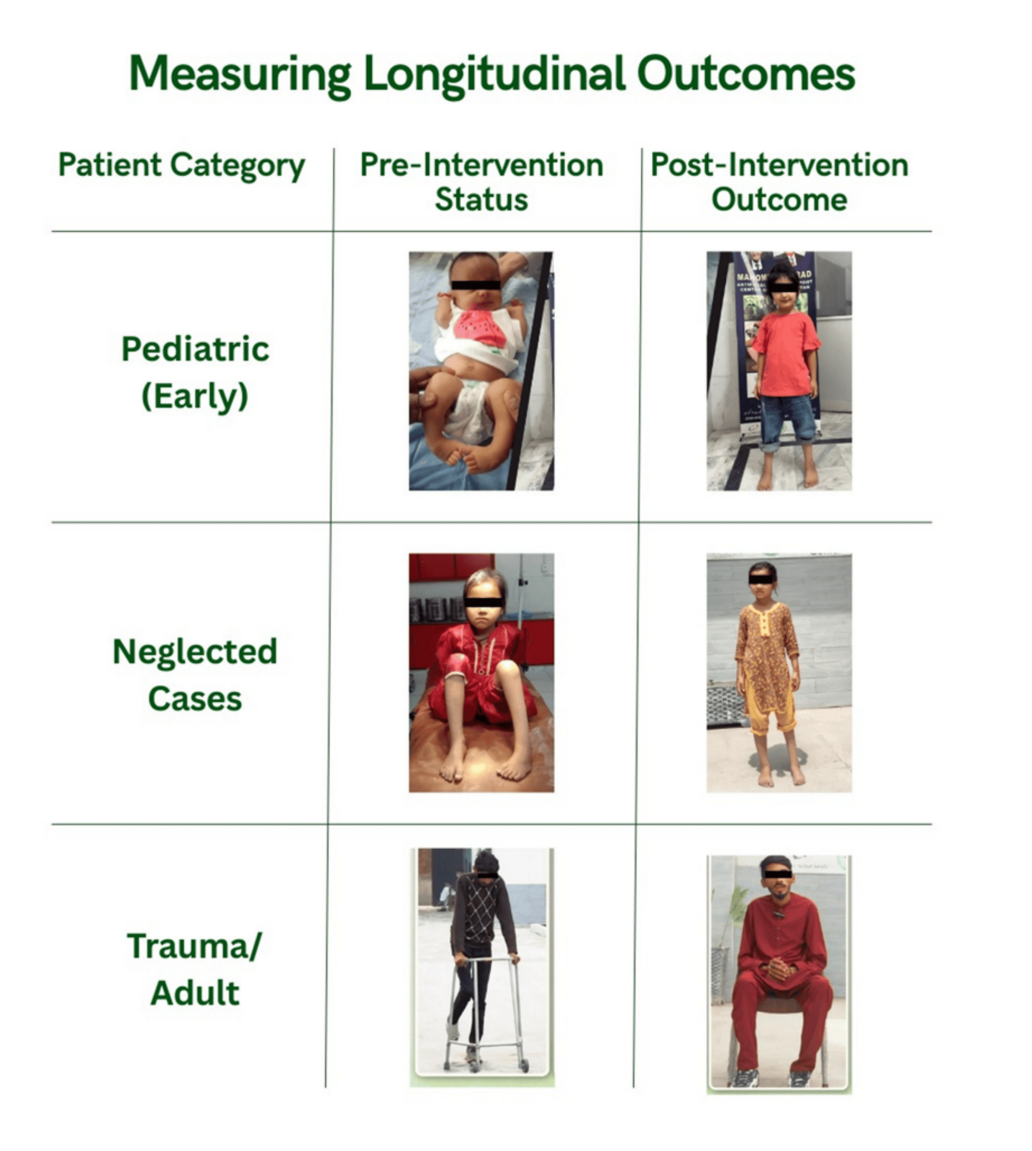
Functional outcomes across patient categories following longitudinal intervention. This figure summarizes observed improvements in correction status, ambulation, and functional mobility across patients receiving structured care. Findings are based on clinical follow-up and program-level outcome assessment. Image credit: Image created using Canva (Canva Pty Ltd., Sydney, Australia). Written informed consent was obtained from all patients for publication of this case report and accompanying images.

Over time, more than 2,000 patients entered care pathways at the center. Given that each patient required an average of 8 to 10 visits during the casting and bracing phases, cumulative patient encounters exceeded 20,000. Program-level outcome data indicate that approximately 90% of patients achieved plantigrade correction, with a relapse rate of approximately 40%, largely associated with compliance challenges. Tenotomy was required in approximately 90% of patients, while surgical intervention was limited to a smaller subset of cases, primarily among neglected deformities. Follow-up adherence improved over time with the implementation of structured follow-up systems. The average follow-up duration during the bracing phase was approximately three to four years, and brace compliance was observed in approximately 80% of patients. Functional outcomes included independent ambulation and school reintegration in the majority of patients, along with substantial improvement in pain-free mobility. Functional outcomes were observed across patient categories and are summarized in Figure 3.

Financing model and sustainability

**Figure 4:**
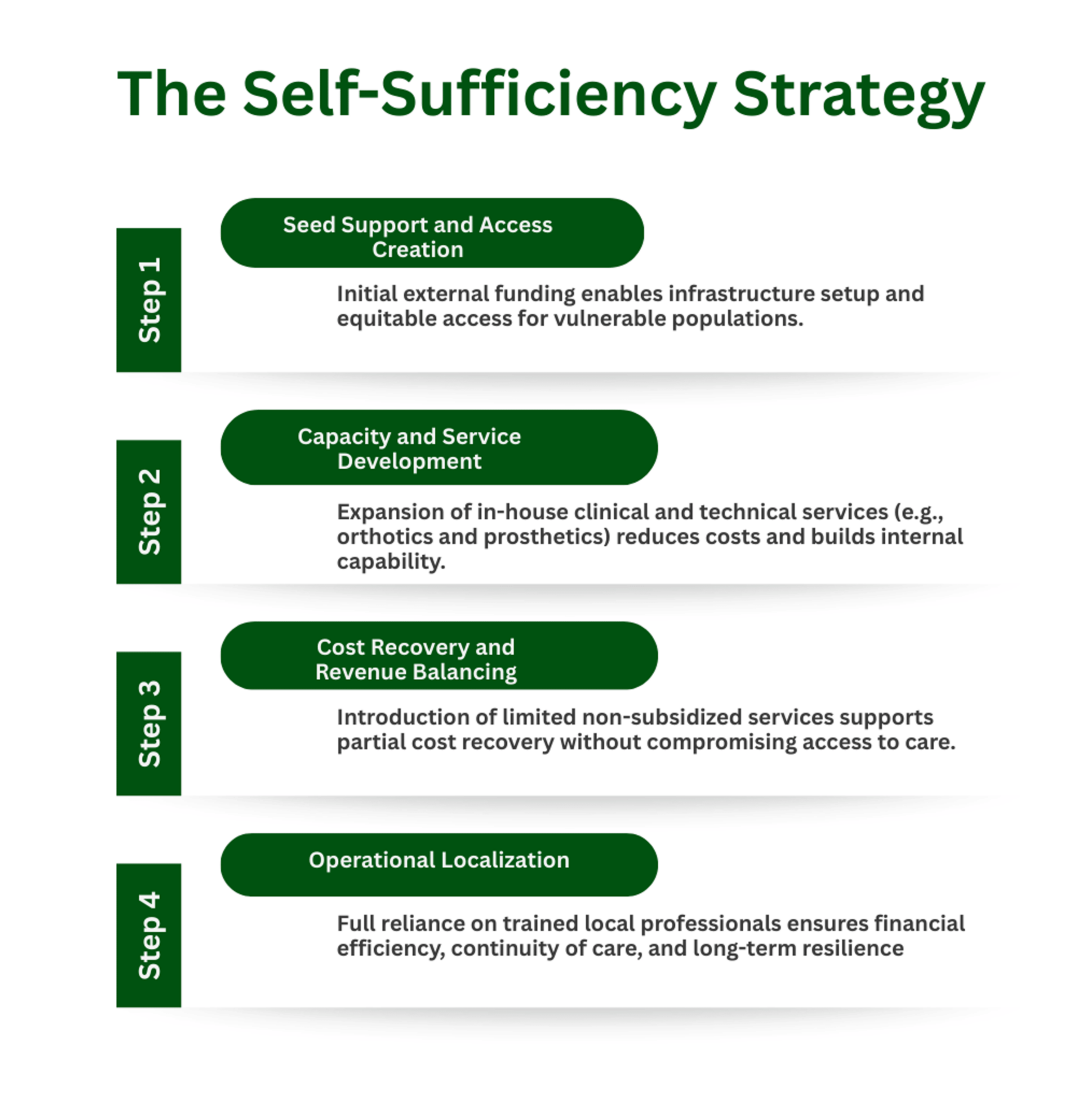
Financial sustainability pathway of the clubfoot rehabilitation program. This figure outlines the transition from donor-supported initiation to a hybrid cost-recovery model, including service-based revenue generation and local capacity development. The model reflects the program’s operational experience and financial evolution. Image credit: Image created by the authors using Canva (Canva Pty Ltd., Sydney, Australia).

Financial sustainability was achieved through a staged transition from donor-supported initiation to a hybrid social enterprise model (Figure 4). Early seed funding established infrastructure and operational stability. Subsequent investment in in-house prosthetic and orthotic fabrication reduced costs and enabled partial cost recovery. Workforce localization further reduced recurring expenditures and strengthened institutional continuity.

## Discussion

The Gujrat model aligns with global evidence demonstrating that the Ponseti method is highly effective in low-resource settings when delivered within structured systems. Long-term follow-up studies have shown success rates exceeding 90% in idiopathic clubfoot when adherence to bracing protocols is maintained [3,4]. Svehlik et al. demonstrated superior long-term functional outcomes and reduced stiffness in Ponseti-treated patients compared with surgically managed cohorts [3]. Similarly, systematic reviews have confirmed that Ponseti-based programs are cost-effective and scalable in LMIC contexts [5].

However, multiple studies emphasize that relapse rates increase significantly when longitudinal follow-up and brace compliance systems are weak [6]. Manzoor et al. reported relapse rates in Pakistan associated with inconsistent brace adherence and irregular follow-up [1]. The Gujrat model addressed this challenge by embedding structured follow-up and case management systems within a locally staffed framework, thereby strengthening treatment continuity.

A recurring limitation in many LMIC clubfoot programs is dependence on international surgical missions, which often deliver episodic care without building sustained capacity [7]. In contrast, capacity-building approaches centered on local workforce training have been associated with improved continuity, cultural appropriateness, and long-term sustainability [8]. The Gujrat experience reinforces this principle by demonstrating that local multidisciplinary teams can maintain quality standards through structured institutional partnerships rather than external dependency.

Financial vulnerability is another common barrier to sustained clubfoot services. Studies examining rehabilitation models in resource-constrained settings highlight the importance of service diversification and social enterprise strategies to mitigate donor dependency [9]. Initial program development was supported through donor-based funding, which enabled infrastructure establishment before transitioning toward a hybrid cost-recovery model. The hybrid financing approach described in this report mirrors these findings, illustrating that in-house fabrication and cost recovery mechanisms can strengthen institutional resilience.

Outcomes in this program were primarily assessed through routine clinical follow-up and programmatic observation rather than standardized, validated instruments, which represents an inherent limitation of the report. Importantly, the impact of structured clubfoot programs extends beyond orthopedic correction. Research has demonstrated significant improvements in quality of life, educational participation, and socioeconomic integration following successful treatment [10]. The functional transitions observed in this program, including workforce reintegration and independent ambulation, are consistent with these broader developmental outcomes. The inclusion of program-level quantitative indicators, such as treatment success rates, relapse rates, and follow-up adherence, strengthens the validity of this model and supports its functional effectiveness in real-world settings. However, variability in data completeness limits the ability to perform detailed statistical analysis.

While this report is descriptive and does not include prospective comparative analysis, its longitudinal operational data support the feasibility of phased, locally governed system development. Future research may benefit from formal cost-effectiveness analysis and standardized functional outcome measurement to further validate scalability.

Limitations

This study has several limitations. First, it is a retrospective, descriptive programmatic report without a control or comparator group. Second, although quantitative program-level indicators were incorporated, standardized longitudinal outcome measurement was not uniformly available across all patients, reflecting variability in documentation over the course of program development. Third, potential selection bias may exist, as patients were enrolled through routine clinical service pathways. Finally, the findings are derived from a single-center experience and may have limited generalizability to settings with different healthcare infrastructure and resource availability. Despite these limitations, the report provides valuable insights into the real-world implementation of a structured and sustainable clubfoot rehabilitation model.

## Conclusions

This technical report demonstrates that comprehensive clubfoot rehabilitation in a low-resource setting can be achieved through phased infrastructure development, locally trained multidisciplinary workforce capacity, structured longitudinal follow-up, and gradual transition toward financial self-reliance. Sustainable disability reduction does not require large centralized institutions but rather disciplined sequencing, local ownership, and integrated service delivery. This model represents a potentially replicable and scalable framework that demonstrates feasibility and real-world effectiveness, while requiring further validation through standardized outcome measurement and comparative studies. The Gujrat model provides a replicable framework for similar regions seeking to address neglected clubfoot and may inform broader rehabilitation system strengthening efforts in LMICs.
